# IL-21 and IL-6 Are Critical for Different Aspects of B Cell Immunity and Redundantly Induce Optimal Follicular Helper CD4 T Cell (Tfh) Differentiation

**DOI:** 10.1371/journal.pone.0017739

**Published:** 2011-03-14

**Authors:** Danelle Eto, Christopher Lao, Daniel DiToro, Burton Barnett, Tania C. Escobar, Robin Kageyama, Isharat Yusuf, Shane Crotty

**Affiliations:** Division of Vaccine Discovery, La Jolla Institute for Allergy and Immunology (LIAI), La Jolla, California, United States of America; Agency for Science, Technology and Research - Singapore Immunology Network, Singapore

## Abstract

Cytokines are important modulators of lymphocytes, and both interleukin-21 (IL-21) and IL-6 have proposed roles in T follicular helper (Tfh) differentiation, and directly act on B cells. Here we investigated the absence of IL-6 alone, IL-21 alone, or the combined lack of IL-6 and IL-21 on Tfh differentiation and the development of B cell immunity in vivo. C57BL/6 or IL-21^−/−^ mice were treated with a neutralizing monoclonal antibody against IL-6 throughout the course of an acute viral infection (lymphocytic choriomeningitis virus, LCMV). The combined absence of IL-6 and IL-21 resulted in reduced Tfh differentiation and reduced Bcl6 protein expression. In addition, we observed that these cytokines had a large impact on antigen-specific B cell responses. IL-6 and IL-21 collaborate in the acute T-dependent antiviral antibody response (90% loss of circulating antiviral IgG in the absence of both cytokines). In contrast, we observed reduced germinal center formation only in the absence of IL-21. Absence of IL-6 had no impact on germinal centers, and combined absence of both IL-21 and IL-6 revealed no synergistic effect on germinal center B cell development. Studying CD4 T cells in vitro, we found that high IL-21 production was not associated with high Bcl6 or CXCR5 expression. TCR stimulation of purified naïve CD4 T cells in the presence of IL-6 also did not result in Tfh differentiation, as determined by Bcl6 or CXCR5 protein expression. Cumulatively, our data indicates that optimal Tfh formation requires IL-21 and IL-6, and that cytokines alone are insufficient to drive Tfh differentiation.

## Introduction

B cell immunological memory consists of long-lived memory B cells and plasma cells, which are the basis for the function and success of almost all human vaccines in use [1]. Memory B cells and long-lived plasma cells are generated within germinal centers (GCs) of secondary lymphoid organs after T-dependent interactions, and the presence of CD4 T cells is essential for GC formation [2], [3]. T follicular helper (Tfh) cells are the CD4 effector subset required to provide B cell help [4], [5], [6], [7], [8].

Tfh were originally identified through their high expression of CXCR5 [9], [10], [11], a chemokine receptor normally found on B cells, which allows these cells to migrate to the B cell follicle [12], [13], [14]. These cells are distinguished from other CD4 subsets by the upregulation of several additional surface molecules including inducible costimulatory molecule (ICOS), CD40L, PD-1, and BTLA [4], [15], [16], [17], [18], [19]. The recent identification of Bcl6 as a master transcriptional regulator of Tfh differentiation [4], [5], [6] and demonstration that Tfh were required for GC formation [4], [5], [6], [7] firmly established Tfh as their own distinct CD4 effector subset.

How Tfh differentiation occurs is currently unresolved. There are currently several proposed models of Tfh development, which center on the cell types involved, the putative mechanisms of Bcl6 induction, and the kinetics of the process [8]. One model proposes that direct induction of Bcl6 via cytokines is sufficient to generate the Tfh subset [5], [7]. A second model suggests that multiple interactions, including B cells, are required for Tfh differentiation [20]. Additional studies have been needed to test these models in detail.

When considering factors controlling Tfh differentiation, it must be done in the context of the knowledge that cytokines are essential for generating many of the known CD4 T cell subsets (Th1, Th2, Th17 and iTreg). Therefore, it is likely that cytokines contribute to Tfh differentiation. Nevertheless, there have been numerous conflicting findings in the literature on this topic. The primary candidate cytokines for Tfh differentiation have been IL-6 and IL-21 [5], [7], [21], [22]. Tfh secrete high levels of IL-21 [7], [16], [17], [23], and work from several laboratories has indicated that IL-21 can affect Tfh differentiation and function [7], [22]. Importantly, multiple labs have found that the lack of either IL-21 alone [24], [25], [26], [27] or IL-6 alone [27], [28] did not substantially impact development of Tfh in vivo in the context of protein immunizations or viral infections. Additionally, IL-21 expression is not restricted to Tfh, as other CD4 Th subsets can produce IL-21 [21], [29], [30], [31], [32]. IL-6 induces IL-21 production [21], [29], [33]. Th17 can be differentiated in vitro from naïve CD4 T cell cultures in the presence of IL-6 and TGFβ, or IL-21 and TGFβ, via a STAT3 dependent pathway [29], [31]. A major potential confounding factor for delineating the role of IL-21 in Tfh differentiation is that IL-6 also primarily signals through STAT3 [28], [34], and may compensate for the lack of IL-21.

Given the varied observations regarding the role of IL-21 and IL-6 in Tfh differentiation, we endeavored to examine the function of these cytokines in greater detail in vitro, and in vivo by studying mice lacking both IL-21 and IL-6. We also further characterized the independent and cooperative nature of IL-21 and IL-6 on B cell immunity.

## Materials and Methods

### Mice

C57BL/6J (B6) and IL-6^−/−^ [35] mice were purchased from Jackson Laboratory (Bar Harbor, ME). IL-21^−/−^ mice were generated as described [24] and obtained from the Zajac lab (University of Alabama). IL-21^−/−^ mice were backcrossed for greater than 10 generations on the B6 background. Whole-genome SNP analysis (Illumina, Golden Gate, SD), verified IL-21^−/−^ mice were >99% B6 by SNP analysis (data not shown). IL-21^−/−^, IL-6^−/−^, and OTII TCR transgenic mice were bred and maintained at the La Jolla Institute for Allergy and Immunology under specific pathogen-free conditions. All animal experiments were conducted on mice 6-12 weeks of age in accordance with the American Association for Laboratory Animal Science on the care and use of animals, and an animal study protocol (AP006-SC1-0809) approved by the La Jolla Institute for Allergy and Immunology Institutional Animal Care and Use Committee.

### In vitro cultures

Total CD4 T cells were isolated from whole splenocytes by negative selection using magnetic beads (Miltenyi). CD4 T cell purity was greater than 95%. Naïve CD4 T cells were further purified by sorting, excluding NKT cells and Tregs (CD4^+^CD44^−^NK1.1^−^CD25^−^) on a FACSAria (BD Biosciences). Cells were resuspended in D-10 (DMEM supplemented with 10% fetal calf serum (FCS), 2 mM GlutaMAX (Gibco), 100 U/mL penicillin/streptomycin (Gibco)) +10 ng/mL hIL-2+50 µM beta-mercaptoethanol (β-ME). Some cultures also included 2 ng/mL IL-7. Cells were stimulated in 24-well plates coated with 8 µg/mL anti-CD3 and 8 µg/mL anti-CD28 (BioXCell). For in vitro differentiated cultures, additional cytokines and blocking antibodies were added at this time, and cells were cultured at 37°C for 72 h: Th0 (forced neutral), 10 µg/mL anti-IFNγ, anti-IL-4, and anti-TGFβ; Th1, 10 ng/mL IL-12+10 µg/mL anti-IL-4 and anti-TGFβ; Th2, 10 ng/ml IL-4+10 µg/mL anti-IFNγ and anti-TGFβ; Th17, 5 ng/ml TGFβ +20 ng/ml IL-6 +10 µg/ml anti-IFNγ and anti-IL-4; Th0+IL-6, 10 ng/ml IL-6+ Th0; Th0+IL-21, 10 ng/ml IL-21+ Th0. In some instances, naïve CD4 T cell cultures were transferred after 24 h onto fresh anti-CD3 coated plates. For the polarizing conditions, all neutralizing cytokine mAbs were purchased from BioXCell, and all recombinant cytokines, except IL-21 (R&D Systems), were purchased from Peprotech. To block IL-21 signaling in vitro, 5 ng/mL IL-21R-Fc (R&D Systems) or 5 µg/ml neutralizing IL-21 mAb (clone FFA21, eBioscience) was added to Th0+IL-21 cultures; to block IL-6 signaling in vitro, 10 µg/ml anti-IL-6 (BioXCell) was added to Th0+IL-6 cultures. CD4 differentiation was assessed after a total of 72 h in culture. After the initial 72 h stimulation, CD4 T cells were transferred into new 24-well plates containing fresh D-10+ IL-2 + β-ME and split as needed. For flow cytometry analysis, live B220^−^CD44^hi^CD4^+^ cells were gated. Normally greater than 80% of stimulated CD4 T cells were CD44^hi^ at these time points.

### Adoptive transfers, immunizations and infections

For viral infections, mice were infected with 1×10^5^ plaque forming units of LCMV Armstrong by intraperitoneal (i.p.) injection. For in vivo antibody treatments, mice were pre-treated with antibody 24 h prior to LCMV infection, and given several additional doses throughout the course of the acute infection. Mice initially received 0.5 mg neutralizing αIL-6 (clone MP5-20F3, rat IgG1; BioXCell) or isotype mAb (anti-rat IgG1; BioXCell), and subsequently given 0.25 mg mAb (i.v.) every other day following the initial dose through the end of the experiment.

For primary protein responses, mice were immunized (i.p.) with 100 µg alum-precipitated NP-Ova (Sigma; Biosearch Technologies) in PBS. Briefly, NP_13_-Ova was dissolved in an equal volume of PBS containing 10% KAl(SO_4_)_2_ (Sigma), and the pH was adjusted to 6.5–7.0 with NaOH. OTII TCR transgenic CD4 T cells were isolated by negative selection using magnetic beads (Miltenyi), and 250,000 OTII cells were transferred intraveneously to host recipients by retro-orbital injections.

### Flow cytometry

Single cell suspensions of whole spleens were obtained by gentle mechanical disruption and ACK lysis (Gibco). Surface staining for flow cytometry used monoclonal antibodies to SLAM (CD150, Biolegend), CD4, CD8, CD44, CD62L, IgD, CD45.1 (eBiosciences), PD-1, FAS, B220, GL7, biotinylated CD138 (BD Pharmingen), and FITC-labeled peanut agglutinin (PNA, Vector Laboratories). The majority of surface stains were done for 30 min at 4°C in FACS buffer (PBS +0.5% BSA +0.1% NaN_3_). As previously described [4], CXCR5 was detected using purified anti-CXCR5 (BD Pharmingen), followed by biotinylated anti-rat IgG (Jackson ImmunoResearch Laboratories), and PE- or APC-labeled streptavidin (eBioscience) in FACS buffer supplemented with 2% FCS and 2% normal mouse serum. Following surface staining, intracellular Bcl6 was detected using a directly conjugated mAb against human Bcl6 that cross-reacts with murine Bcl6 (clone K112-91; BD Pharmingen) using the FOXP3 nuclear staining protocol (eBioscience). For intracellular cytokine staining, splenocytes were stimulated with 400 ng/mL PMA and 150 ng/mL ionomycin in the presence of brefeldin A (BFA) for 4 h, followed by surface staining. Intracellular cytokine staining was performed with directly conjugated mAb against IFN-γ, IL-4, or IL-17 (eBioscience). IL-21 was detected using an IL-21R-Fc chimeric protein (R&D Systems) followed by PE-labeled anti-human IgG (Jackson ImmunoResearch). Samples were acquired on an LSRII containing DIVA software (BD Biosciences), and analyzed with FlowJo software (Treestar, Inc).

### ELISA

Anti-LCMV IgG was quantified from mouse serum by ELISA 8 days following LCMV infection as previously described [36]. LCMV cell lysate was used as the capture antigen to coat 96-well Polysorp microtiter plates (Nunc). IL-21 was quantitated from the supernatant of in vitro differentiated CD4 T cells. Day 7 polarized CD4 T cells were stimulated overnight with 400 ng/mL PMA and 150 ng/mL ionomycin. 96-well Maxisorp microtiter plates (Nunc) were coated with an anti-mouse IL-21 affinity purified antibody (goat IgG, R&D Systems) as the capture antibody. Following the primary incubation, samples were treated with biotinylated goat anti-mouse IgGγ (Southern Biotech) to detect anti-LCMV IgG, or biotinylated goat anti-mouse IL-21 (R&D Systems) to detect IL-21. Plates were subsequently incubated with HRP conjugated Avidin D (Vector Laboratories) and visualized using OPD (o-phenylenediamine dihydrochloride; Sigma) substrate.

### Quantitative real-time PCR

RNA was extracted with an RNeasy micro kit (Qiagen), including Qiashredder and on-column digestion of genomic DNA. cDNA synthesis was performed using SuperScript II Reverse Transcriptase (Invitrogen) with random hexamer primed reactions. Quantitative real-time PCR (qPCR) reactions were performed in triplicate using iTaq SYBR Green Supermix with ROX (Bio-Rad) on a Roche LightCycler 480 (Roche). Primers were as described [4]. G6PDH was used as the reference gene for normalization.

### Statistical analysis

All statistical tests were performed using Prism 5, and p values were calculated using two-tailed, unpaired Student t tests, with a 95% confidence interval. Error bars represent the SEM. P<0.05 =  *, P<0.005 =  **. P<0.0005 =  ***.

## Results

### IL-21 is produced by many CD4 T effector subsets

**Figure 1:**
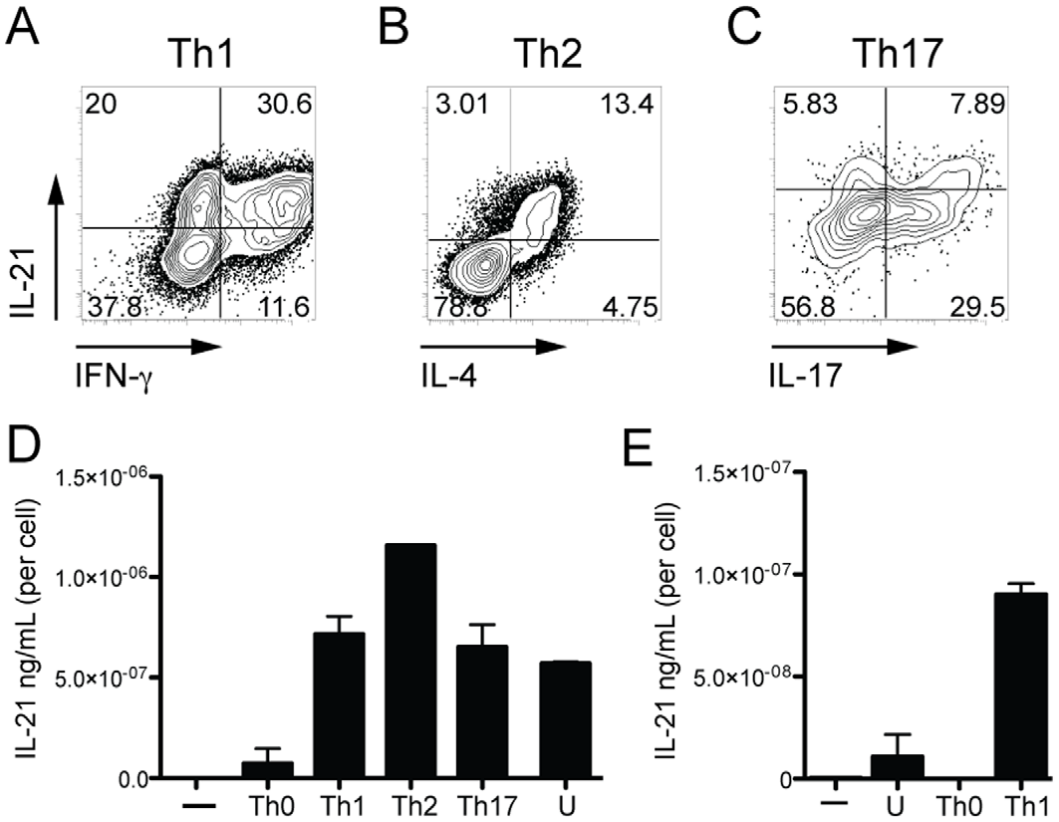
IL-21 can be produced by Th1, Th2 or Th17 cell in vitro. (**A–D**) Magnetic bead purified total CD4 T cells were stimulated with plate-bound αCD3 plus αCD28 without cytokine blocking mAbs (unbiased, U), or in the presence of αIFNγ + αIL-4 (Th0), IL-12 + αIL-4 (Th1), IL-4 + αIFNγ (Th2), or IL-6/TGFβ + αIFNγ & αIL-4 (Th17). Day 7 CD4^+^B220^−^ gated in vitro cultured cells were stained by ICS after 4 h stimulation in PMA/ionomycin. Representative dot plots of IL-21 and the respective cytokine associated with each subset, (**A**) IFNγ (Th1), (**B**) IL-4 (Th2), or (**C**) IL-17 (Th17). (**D**) IL-21 was measured by ELISA from day 7 cells that had been washed, and then restimulated overnight in the presence of PMA/ionomycin. IL-21 was normalized to cell number per well. (**E**) Sorted naïve CD4 T cells (CD4^+^CD44^−^NK1.1^−^CD25^−^) were cultured under unbiased (U), Th0 or Th1 conditions. ELISA analysis of IL-21 production from day 7 cultured CD4 T cells restimulated overnight with PMA/ionomycin (normalized to cell number per well). “—”  =  no stim. Data are representative of ≥2 independent experiments.

Some activated CD4 T cells can produce high amounts of IL-21 [21], [30], [31], [32]. IL-21 production by Th1, Th2, and Th17 polarized CD4 T cell cultures was investigated by intracellular staining for IL-21 protein. Splenic CD4 T cells from C57BL/6 mice were isolated by magnetic bead negative selection, and stimulated with αCD3 and αCD28 in the presence or absence of exogenous cytokine and appropriate neutralizing antibodies. We observed that CD4 T cells cultured for 7 days under Th1 polarizing conditions co-produced high levels of IFNγ and IL-21 (Figure 1A). Under Th2 conditions CD4 T cells co-produced IL-4 and IL-21 (Figure 1B). CD4 T cells cultured under Th17 conditions co-produced IL-17 and IL-21 (Figure 1C), as has been widely reported [21], [29], [30], [31]. To confirm our flow cytometry results, IL-21 from culture supernatants was measured by ELISA, and normalized to cell numbers. After 7 days in culture, CD4 T cells were stimulated overnight in the presence of PMA and ionomycin. Supernatants from CD4 T cells cultured under forced neutral conditions (Th0) produced little IL-21 compared to CD4 T cells cultured under Th1, Th2, and Th17 conditions (Figure 1D). Of note, magnetic bead purified CD4 T cells stimulated with αCD3 and αCD28 alone, without blocking monoclonal antibodies (Unbiased, U), produced large amounts of IL-21 (Figure 1D). Collectively, we interpreted those results to mean that IL-21 production by memory CD4 T cells was sufficient to prime autocrine IL-21 production by activated naïve CD4 T cells.

### IL-21 production induced in naïve CD4 T cells

**Figure 2:**
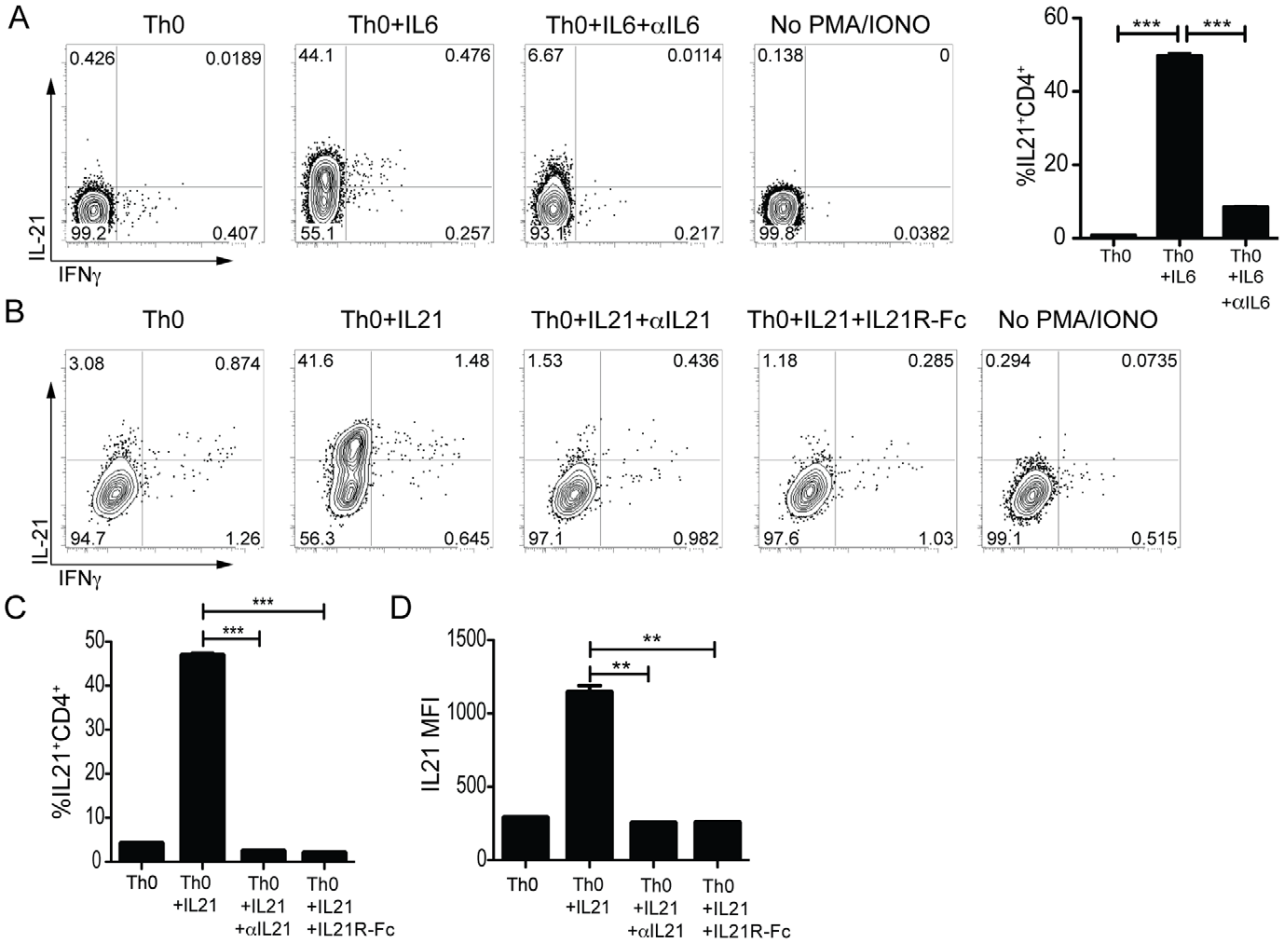
In vitro stimulation of purified naïve CD4 T cells with IL-6 or IL-21 drives high IL-21 expression. (**A–D**) Naïve sorted CD4 T cells (CD4^+^CD44^−^NK1.1^−^CD25^−^) were stimulated for 3 days on αCD3/αCD28 coated plates in the presence of αIFNγ, αIL-4, and αTGFβ (Th0) ± IL-6 or IL-21. Reagents to block IL-21 (5 µg/ml) or IL-6 (10 µg/ml) signaling were also added at this time where indicated. IL-21R-Fc intracellular staining was used to identify IL-21 producing cells after 4 h of PMA/ionomycin stimulation. (**A**) Flow cytometry analysis of IL-21 and IFNγ production in day 7 Th0, Th0 + IL-6, and Th0 + IL-6 + αIL6 polarized CD4 T cell cultures. Cells were gated on B220^−^CD44^hi^ CD4 T cells. *** P<0.0005. (**B–D**) FACS analysis of IL-21 and IFNγ production in day 3 Th0 + IL-21± αIL-21 or IL-21R-Fc differentiated CD4 T cells. (**B**) Representative plots (B220^−^CD44^hi^ CD4 T cells). (**C**) Quantitation of IL-21 production or (**D**) IL-21 MFI. ** P<0.003, *** P<0.0003. Data are representative of ≥2 independent experiments for each condition with duplicate samples. MFI, mean fluorescence intensity.

We then examined purified naïve T cell cultures under more stringently controlled conditions. Naïve, FACS sorted CD4 T cells, excluding Tregs and NKT cells, (CD44^lo^CD25^−^NK1.1^−^) did not produce IL-21 upon TCR stimulation alone (Figure 1E), or under neutral (Th0) conditions (Figure 1E & 2A–B). Therefore, sorted naïve CD4 T cells were used for all subsequent experiments. Addition of IL-6 to naïve CD4 T cell cultures (Th0 + IL6) dramatically increased the frequency of IL-21 producing CD4 T cells compared to Th0 cultures (44% vs. 0.5%; P = 0.0002; Figure 2A). IL-21 production was directly mediated through IL-6 signaling, as the presence of a neutralizing anti-IL-6 monoclonal antibody (mAb) in the culture led to a large reduction in IL-21 expression levels (6%; P = 0.0004).

High IL-21 expression could also be induced by the addition of IL-21 (42% vs. 3%; Figure 2B-D) [7], [21]. This effect was directly mediated through IL-21 signaling, as the addition of soluble IL-21 receptor (IL-21R-Fc) reduced the generation of IL-21 producing CD4 T cells in Th0 + IL-21 cultures to background levels (2%; P<0.0002; Figure 2B-D)[21]. We also screened anti-IL-21 mAbs for IL-21 neutralization activity and identified a clone with potent activity. Addition of neutralizing anti-IL-21 to Th0 + IL-21 cultures completely blocked the differentiation of IL-21 producing CD4 T cells (P = 0.0002; Figure 2B–D). The addition of IL-6 or IL-21 to naïve CD4 T cell cultures specifically induced IL-21 secretion, as IFNγ production was not upregulated (Figure 2A–B). Thus, we confirm and extend the finding that IL-21 expression is strongly induced in CD4 T cells by IL-6 or IL-21.

### High IL-21 production does not instruct Tfh differentiation in vitro

**Figure 3:**
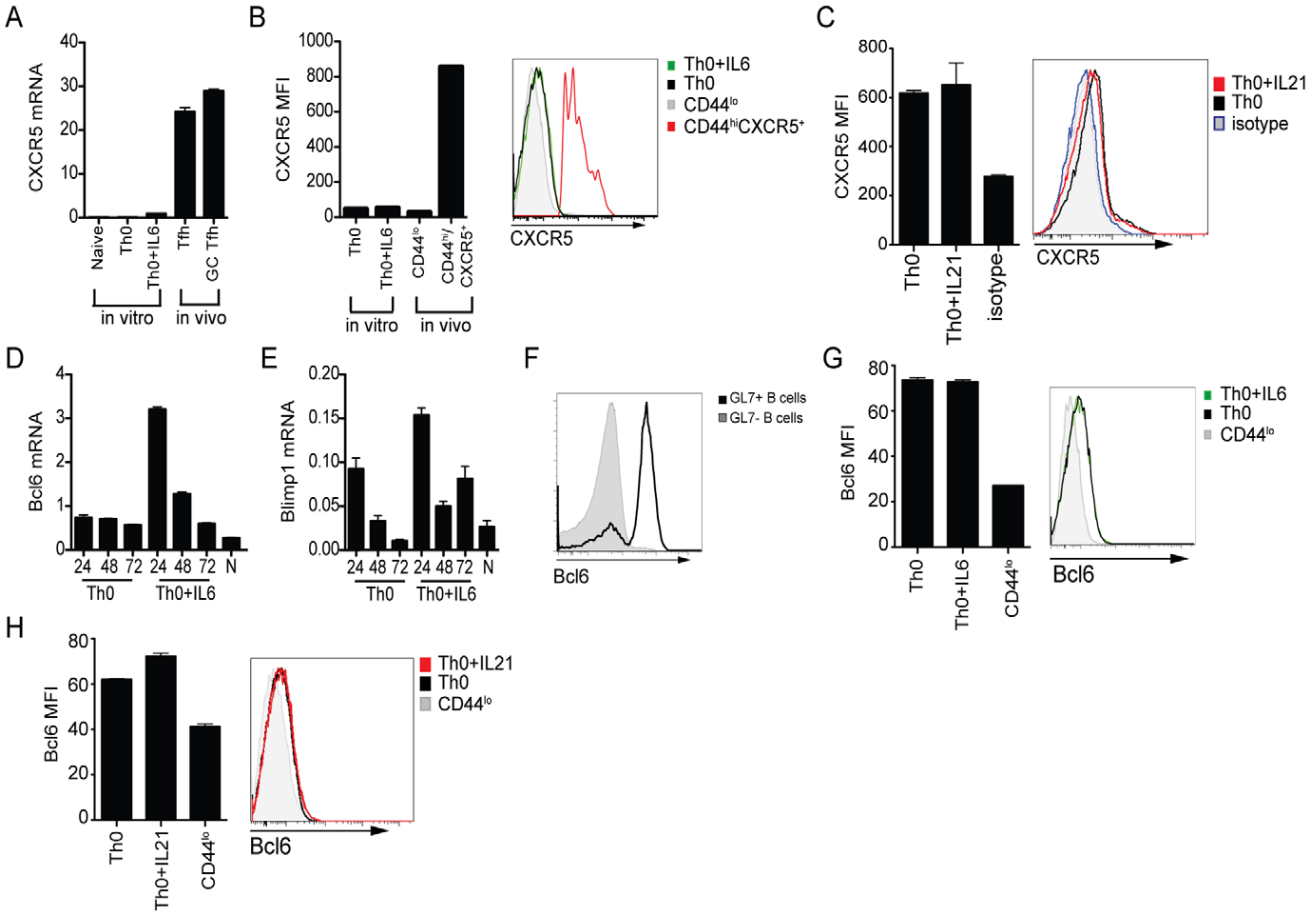
High IL-21 production in CD4 T cell cultures does not instruct Tfh differentiation. Naïve sorted CD4 T cells were stimulated for 72 h on αCD3 plus αCD28 coated plates in the presence of αIFNγ, αIL-4, and αTGFβ (Th0) ± IL-6 or IL-21. (**A**) CXCR5 mRNA expression normalized to a housekeeping gene, G6PDH, in naïve (CD4^+^CD44^lo^), day 3 in vitro polarized CD4 T cell cultures (Th0 or Th0 + IL-6), or OTII CD4 T cells from day 8 NP-OVA plus alum in vivo immunized mice (CD44^hi^CXCR5^+^GL7^−^ Tfh or CD44^hi^CXCR5^+^GL7^+^ GC Tfh). (**B**) Quantitation of CXCR5 mean fluorescence intensity (MFI) in day 3 cultures (Th0 or Th0 + IL-6) or CD4 splenocytes (naïve, CD44^lo^; CXCR5^+^, CD44^hi^), and histogram. (**C**) CXCR5 analysis in 72 h Th0 (black) or Th0 + IL-21 (red) cultures. Th0 and Th0 + IL-21 cultures stained with isotype mAb (blue). (**D–E**) Timecourse of Bcl6 (**D**) and Blimp-1 (**E**) mRNA expression normalized to a housekeeping gene, G6PDH, in Th0 or Th0 + IL-6 CD4 T cell cultures. (**F**) FACS analysis of Bcl6 protein expression in germinal center B cells (GL7^+^B220^+^) versus non GC B cells (GL7^−^B220^+^) in splenocytes 8 days following LCMV infection. (**G**) Bcl6 protein expression in day 3 in vitro differentiated CD4 cultures (Th0 or Th0 + IL-6) versus freshly isolated naïve CD4 splenocytes (CD44^lo^). Left, quantitation of Bcl6 MFI. Right, Bcl6 histogram. (**H**) Bcl6 analysis in 72 h Th0 (black) or Th0 + IL-21 (red) cultures. Naïve splenocytes are stained for comparison (CD44^lo^, gray). Data are representative of ≥2 independent experiments with duplicate samples.

While the addition of IL-6 or IL-21 to naïve CD4 T cell cultures induced IL-21 expression, we wondered if these conditions generated true Tfh cells in vitro. Tfh are defined by high CXCR5 expression and expression of the Tfh transcription factor Bcl6. Tfh differentiation requires Bcl6 expression [4], [5], [6]. We therefore next examined the role of IL-6 and IL-21 in driving Tfh development in CD4 T cells. We first compared CXCR5 mRNA levels of sorted naïve CD4 T cells stimulated in the presence or absence of IL-6 versus in vivo Tfh. In vivo OTII Tfh were generated by protein immunization of C57BL/6 mice that received adoptively transferred OTII cells. There are two stages of Tfh differentiation [8]. CD4 T cells found within the germinal center (GC Tfh) are a more polarized population of Bcl6^+^CXCR5^+^ Tfh CD4 T cells that express the highest level of PD-1, SAP, and GL7 and have enhanced B cell help capabilities [8], [16], [17], [37]. At day 8 after NP-Ova plus alum immunization, OTII Tfh (CXCR5^+^GL7^−^) and OTII GC Tfh (CXCR5^+^GL7^+^) express high levels of CXCR5 mRNA (Figure 3A). CD4 T cells differentiated in the presence of IL-6 (Th0 + IL-6) modestly upregulate CXCR5 mRNA compared to Th0 cultures (P<0.0001; Figure 3A). However, in vitro “Th0 + IL-6” cell CXCR5 mRNA levels were 20-fold lower than in vivo Tfh (Figure 3A). We next assessed CXCR5 protein expression by flow cytometry, and observed negligible CXCR5 protein expression by in vitro Th0 or Th0 + IL-6 cells (Figure 3B). CXCR5 expression on freshly isolated naïve (CD44^lo^) or CD44^hi^CXCR5^+^ CD4 splenocytes is shown for comparison (Figure 3B). CXCR5 surface expression by in vitro cultured cells was also unaffected by the addition of IL-21 (Th0 + IL-21; Figure 3C). Together, these data indicate that IL-21 and CXCR5 expression are independently regulated in CD4 T cells.

We next examined whether IL-21 modulates the expression of Bcl6. Expression levels of Blimp-1 were examined in parallel, as Blimp-1 is a potent antagonist of Bcl6 that inhibits Tfh differentiation [4], [38]. Previous studies have shown that IL-6 and IL-21 can induce or sustain Bcl6 mRNA in CD4 T cells or B cells [5], [7], [26], [39]. In addition, Blimp-1 can also be induced by IL-21 [40]. As the expression of these transcription factors is intimately tied to Tfh differentiation [4], we assessed Bcl6 and Blimp-1 mRNA expression at several early timepoints after stimulation of naïve CD4 T cells in vitro. Th0 cultures expressed low levels of Bcl6 mRNA, similar to naïve CD4 T cells, which did not change over the course of the experiment (Figure 3D). Addition of IL-6 to the Th0 cultures led to a sharp increase in Bcl6 mRNA at 24 h post-stimulation, which quickly tapered off to Th0 levels by 72 h (Figure 3D), similar to one report [5] though different than another report that saw no Bcl6 induction [28]. IL-6 also led to an increase in Blimp-1 mRNA at 24 h (Figure 3E), and Blimp-1 levels appeared to be better maintained than Bcl6 at the later timepoints (Figure 3D–E). Blimp-1 expression after IL-6 treatment was significant, as the 6-fold increase at 24 h and 3-fold increase at 72 h was similar to the 5-fold differences seen between in vivo non-Tfh and naive CD4 T cells studied contemporaneously [17]. Thus, IL-6 alone does not selectively induce Bcl6 mRNA over Blimp-1 mRNA in CD4 T cells.

Post-transcriptional regulation can strongly impact Bcl6 expression, and can result in substantial discrepancies between Bcl6 mRNA and protein expression [20], [41], [42]. Therefore, the ability to assess Bcl6 protein at the single cell level was essential for a fuller understanding of Bcl6 regulation in CD4 T cells. We established an intracellular flow cytometry stain to detect Bcl6 protein with a new monoclonal antibody. Bcl6 staining conditions were first optimized by comparing naïve versus germinal center B cells (GL7^−^ vs. GL7^+^; Figure 3F). We then examined Bcl6 protein expression in 72 h CD4 T cell cultures. The addition of IL-6 did not increase Bcl6 protein expression above basal Th0 levels (Figure 3G). A slight increase in Bcl6 expression was observed with the addition of IL-21 to Th0 cultures (Figure 3H). However, Bcl6 upregulation in the Th0 + IL-21 cells did not correlate with enhanced CXCR5 surface expression. Collectively, our results demonstrate that TCR stimulation plus IL-6 or IL-21 cytokine alone is not enough to drive Tfh differentiation of purified naïve CD4 T cells in vitro, as neither Bcl6 nor CXCR5 protein expression were significantly induced.

### Combined IL-6 and IL-21 deficiency in vivo

In order to investigate the roles of IL-6 and IL-21 in Tfh differentiation and the development of B cell immunity, we utilized a murine model. We recently reported that Tfh differentiation and germinal center development were normal in IL-6^−/−^ mice [27]. Others have shown Tfh differentiation to be relatively unaffected in IL-21^−/−^ mice [24], [25], [26], [27]. However, different laboratories have had different results or interpretations [7], [22]. As both cytokines signal through STAT3 [32], [43], we hypothesized that these two cytokines could have redundant, collaborative functions in vivo.

**Figure 4:**
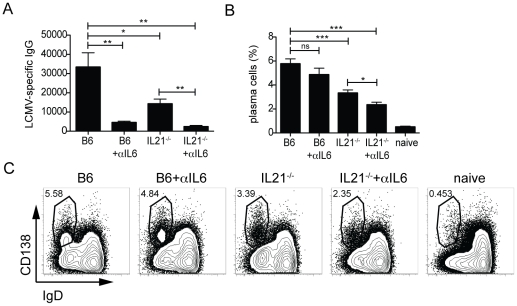
Cooperation of IL-6 and IL-21 for plasma cell generation and antibody responses. C57BL/6 (B6) or IL-21^−/−^ mice treated with αIL-6 or isotype mAb throughout the acute LCMV infection. (**A**) Titers of anti-LCMV IgG in the serum of B6 or IL-21^−/−^ mice ± αIL-6 mAb 8 days following LCMV infection: B6 vs. B6 + αIL-6 (**, P = 0.0016), B6 vs. IL-21^−/−^ (*, P = 0.025), B6 vs. IL-21^−/−^ + αIL-6 (**, P = 0.0016), and IL-21^−/−^ vs. IL-21^−/−^ + αIL-6 (**, P = 0.0012). (**B**) Quantitation of plasma cell development by FACS: B6 vs. IL-21^−/−^ (***, P = 0.0008), B6 vs. IL-21^−/−^ + αIL-6 (***, P<0.0001), and IL-21^−/−^ ± anti-IL-6 (*, P = 0.0143). (**C**) Plasma cell (CD138^+^IgD^−^) development 8 days post-infection, gated on CD19^+^ splenocytes. Data are representative of 3 independent experiments; n = 5–6 mice per group. ns, not significant.

IL-6 deficient mice have a 2–4 fold decrease in early T-dependent virus-specific IgG responses compared to wildtype C57BL/6 (B6) mice following acute infection with LCMV [27]. We determined that the IL-6^−/−^ phenotype could be recapitulated by blocking IL-6 signaling using a neutralizing IL-6 mAb. Treatment of B6 mice with αIL-6 mAb led to a severe loss of day 8 serum LCMV-specific IgG compared with B6 mice given an isotype control mAb (20% of wildtype level; P = 0.0016; Figure 4A). Thus, ablation of IL-6 signaling could be achieved in B6 mice by treatment with neutralizing αIL6 mAb.

### Differential role of IL-6 and IL-21 in development of B cell immunity

Having validated the in vivo IL-6 blockade, we could test the importance of IL-6 alone (αIL-6), IL-21 alone (IL-21^−/−^), or the combined absence of IL-6 and IL-21 (IL-21^−/−^+ αIL-6) for Tfh differentiation and the development of B cell immunity. We examined the impact of these cytokines on the early antiviral antibody response at day 8 after acute LCMV infection. Serum antibody levels were quantitated by endpoint ELISA analysis (Figure 4A). The absence of IL-21 alone (IL-21^−/−^) led to a 50% loss of LCMV specific IgG compared to B6 mice (P = 0.0016) [25], [26], [27], [44], and this loss was magnified in the absence of both IL-21 and IL-6 (7% of wildtype level; P = 0.002; Figure 4A). At day 8, the peak of the plasma cell response [45], we observed a significant decrease in plasma cell (CD138^+^IgD^−^CD19^+^) frequency by flow cytometry in IL-21^−/−^ mice compared to B6 mice (P<0.0008). Interestingly, while antibody levels were substantially reduced in the absence of IL-6, plasma cell development was modestly impacted (6% vs. 5%; B6 vs. B6 + αIL-6; P = 0.2056). The largest reduction in plasma cell frequency was observed in the combined absence of both IL-6 and IL-21 (B6 vs. IL-21^−/−^ + αIL-6; P<0.0001; Figure 4B–C).

Germinal center formation is detectable at day 6 after acute LCMV infection, with a large germinal center response present by day 8 post-infection. The reduction in plasma cell numbers at day 8 in the absence of IL-21 is most likely a reflection of the critical role of IL-21 in driving plasma cell differentiation from germinal center B cells (Figure 4B–C) [25], [26], [46]. IL-21 is a potent STAT3-dependent inducer of Blimp-1 [40], [47], [48]. Thus the combined absence of IL-6 and IL-21 through 8 days post-infection results in a predominantly IL-6 dependent loss of antibody production by the extrafollicular plasma cells (IL-21^−/−^ vs. IL21^−/−^ + αIL-6; P<0.0143; Figure 4B–C), and the absence of germinal center derived plasma cells (IL-21 dependent), resulting in a near complete abrogation of the serum antibody response to the viral infection.

**Figure 5:**
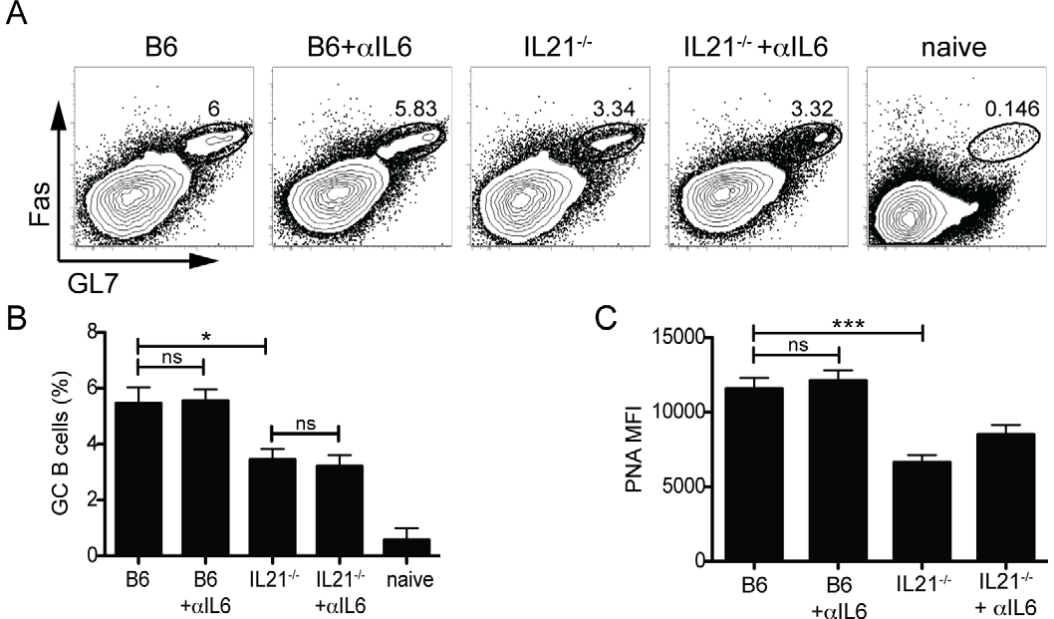
IL-21, but not IL-6, is needed for optimal germinal center B cell development. (**A**) Germinal center B cells (GL7^+^Fas^+^ gated, total B cell gate shown) in C57BL/6 (B6) or IL-21^−/−^ mice treated with anti-IL-6 (αIL6) or isotype mAb. Splenocytes were analyzed 8 days following acute LCMV infection. (**B**) Frequency of germinal center B cells of total B cells. *, P<0.02. (**C**) Quantitation of the PNA mean fluorescence intensity (MFI) in germinal center B cells. ***, P = 0.0003. Data are representative of 3 independent experiments; n = 5–6 mice per group. MFI, mean fluorescence intensity; ns, not significant.

Previous studies have characterized the individual roles of IL-21 or IL-6 on B cells and found that IL-21 has a large impact on germinal center development [25], [26], [35]. We examined what effect the combined absence of both IL-21 and IL-6 would have on germinal center formation (Figure 5). Germinal center B cell (GL7^+^Fas^+^) frequencies were similar in B6 mice treated with either αIL-6 or isotype mAb (Figure 5A–B). However, the absence of IL-21 led to a consistent 50% loss of germinal center B cells compared to B6 mice (P = 0.0233; Figure 5A–B). As previously noted [25], PNA staining intensity is decreased on germinal center B cells in the absence of IL-21 (P = 0.0003; Figure 5C), indicative of reduced GC B cell proliferation. Interestingly, germinal center B cell numbers in IL-21^−/−^ mice were unaffected by the additional loss of IL-6 (Figure 5A–B), as was PNA MFI (Figure 5C). Thus, while IL-21 and IL-6 cooperate in stimulating plasma cell responses, IL-21 has a unique role in enhancing germinal center B cell proliferation, maintenance, and function.

### Optimal Tfh differentiation requires IL-6 or IL-21

**Figure 6:**
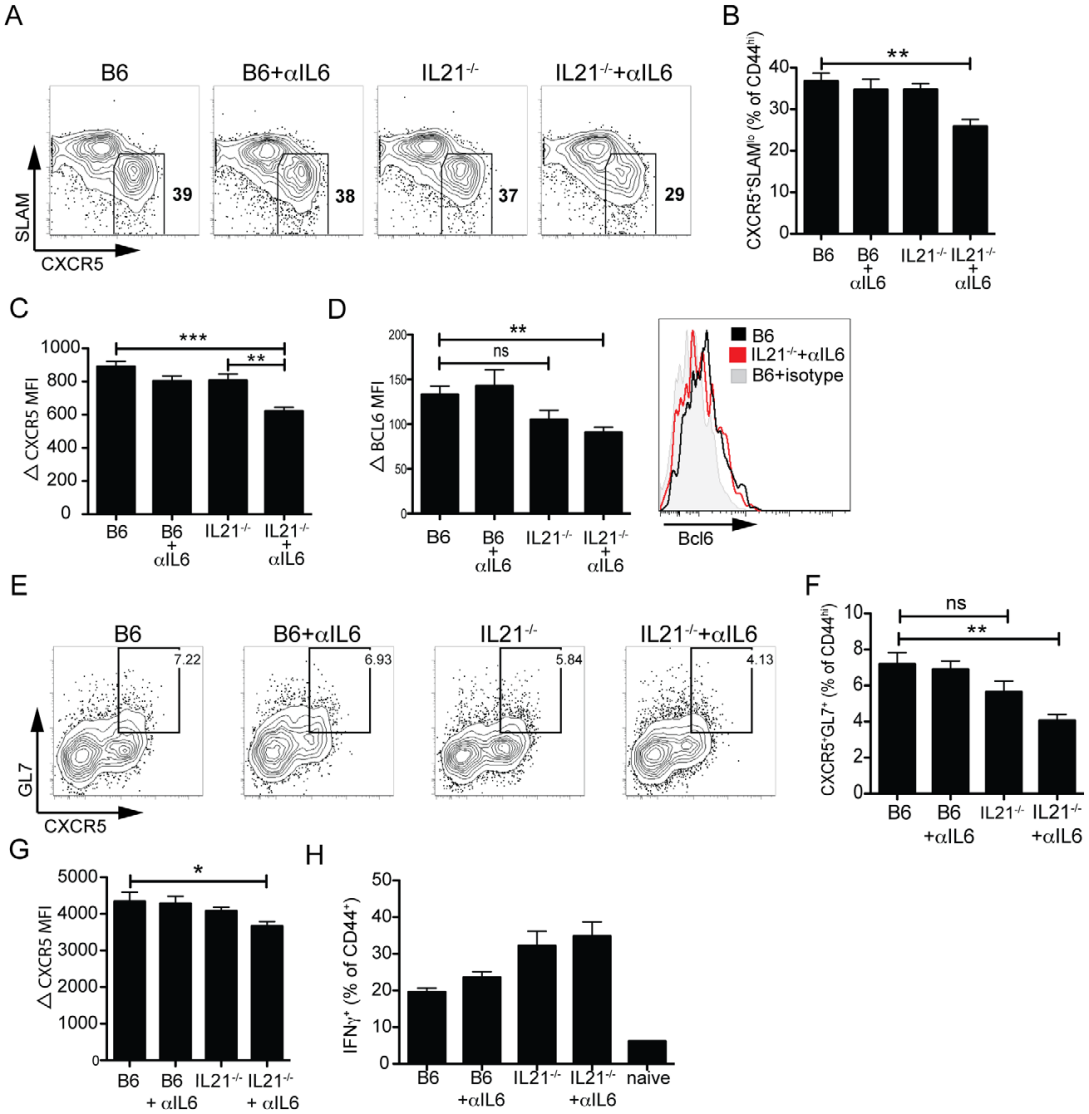
IL-6 or IL-21 is required for optimal Tfh differentiation. (**A–G**) Splenocytes were analyzed 8 days following LCMV infection in C57BL/6 (B6) or IL-21^−/−^ mice ± αIL6. (**A**) Follicular helper T (Tfh) cells (CXCR5^+^SLAM^lo^, boxed) gated on activated (CD44^hi^) CD4 T cells. (**B**) Percentage of Tfh differentiation (from gated Tfh population). **, P = 0.0016. (**C**) Quantitation of ΔCXCR5 mean fluorescence intensity (MFI) gated on Tfh (normalized against CD44^lo^ CD4 T cells). **, P = 0.003. ***, P<0.0001. (**D**) Left, quantitation of Bcl6 protein ΔMFI in Tfh (normalized against CD44^lo^ CD4 T cells). ***, P = 0.0009. Right, histogram overlay shows Bcl6 expression in a representative B6 vs. IL-21^−/−^ + αIL-6 Tfh population (B6, black; IL-21^−/−^ + αIL-6, red; B6 stained with isotype mAb, gray). (**E**) FACS analysis of germinal center CD4 T (GC Tfh) cells (CXCR5^+^GL7^+^, boxed). Gated CD44^hi^ CD4 T cells shown. (**F**) GC Tfh quantitation. **, P = 0.003. (**G**) ΔCXCR5 MFI, gated on GC Tfh and normalized against CD44^lo^ CD4 T cells. *, P = 0.0405. Flow cytometric analysis of intracellular cytokine staining for IFNγ production by CD4 T cells after 4 h stimulation with PMA and ionomycin in the presence of brefeldin A. (**H**) Frequency of IFNγ^+^CD44^hi^ CD4 T cells. Data are representative of ≥2 independent experiments; n = 5–6 mice per group. MFI, mean fluorescence intensity; ns, not significant.

We next examined what effect the combined absence of IL-6 and IL-21 would have on Tfh development (Figure 6). Similar to previous observations [24], [25], [26], [27], we did not observe major differences in the development of Tfh (CD44^hi^CXCR5^+^SLAM^lo^) in the absence of either IL-6 alone [27], [28], or IL-21 alone [24], [25], [26], compared to WT B6 mice (Figure 6A–B). However, the combined absence of both IL-6 and IL-21 resulted in a 25% loss in Tfh generation compared to wildtype mice (P = 0.0016; Figure 6A–B). We also observed a strong correlation between reduced Tfh frequency and CXCR5 protein expression levels within the Tfh population. No difference in CXCR5 protein expression was observed in the absence of IL-6 alone or IL-21 alone (Figure 6C). However, reduced CXCR5 MFI was observed in the combined absence of both IL-6 and IL-21 (B6 vs. IL-21^−/−^ + αIL-6; P<0.0001; Figure 6C). Similar to what we characterized in GC B cells, increased Bcl6 protein expression in Tfh (CXCR5^+^) versus non-Tfh (CXCR5^−^) cells can be observed by FACS (Figure 6D). Compared to B6 controls, the absence of IL-6 (B6 + αIL-6) had no effect on Bcl6 protein expression in the Tfh population (Figure 6D). Although the lack of IL-21 (IL-21^−/−^) did appear to result in lower Bcl6 protein expression, this did not reach statistical significance (P = 0.073; Figure 6D). However, Bcl6 expression was significantly decreased in Tfh generated in the absence of both IL-6 and IL-21 (P = 0.0032, Figure 6D).

We also examined the development of the GC Tfh population in the absence of IL-6, IL-21, or combined absence of both IL-6 and IL-21. GC Tfh (CD44^hi^CXCR5^+^GL7^+^) were observed in all conditions, and the frequency of GC Tfh in the absence of either IL-6 alone or IL-21 alone was similar to B6 mice (Figure 6E-F). However, the combined absence of both IL-6 and IL-21 led to a 40% reduction in GC Tfh frequency compared to B6 mice (P<0.003; Figure 6E–F). CXCR5 fluorescence intensity was quantitated in the GC Tfh subset for the four different conditions, and only in the absence of both IL-6 and IL-21 was a significant reduction in CXCR5 protein observed (P = 0.04; Figure 6G). Collectively, these data demonstrate that optimal Tfh differentiation requires cooperation between IL-6 and IL-21.

In order to determine whether the reduction in Tfh differentiation that we observed in the combined absence of IL-6 and IL-21 was specific to the Tfh subset, we characterized the frequency of antigen specific CD4 T cells. Similar to previous observations [24], [27], [49], the lack of either IL-6 or IL-21 did not lead to any reduction in IFNγ^+^ CD4 T cells, and we did not observe any loss in the combined absence of both IL-6 and IL-21 (Figure 6H). Although both cytokines are reported to negatively regulate IFNγ production [50], [51], IL-21, more so than IL-6, appeared to suppress the generation of non-Tfh antigen specific cells (Figure 6H). Thus, our data suggests that the observed reduction in Tfh in the absence of both IL-6 and IL-21 is specific to the Tfh subset, and not due to a general suppression of antiviral CD4 T cell responses.

## Discussion

This is the first study to characterize cooperative functions for IL-6 and IL-21 in Tfh differentiation and generation of B cell immunity in vivo. IL-21 is known to be a major factor for generation of sustained high affinity antibody responses in vivo [25], [26], [39], [46], consistent with IL-21 being the most potent known inducer of plasma cell differentiation in vitro [48], [52], [53]. In addition, IL-21R^−/−^ and IL-21^−/−^ mice exhibit germinal center defects [22], [44]. B cells expressing IL-21R and IL-21 were recently shown to be important, in a B cell intrinsic manner, for optimal proliferation of germinal center B cells [25], [26]. Given that IL-6 and IL-21 share signaling pathways, and B cells express receptors for both cytokines, we characterized B cell responses in mice deficient for both IL-6 and IL-21 signaling.

The combined loss of both IL-6 and IL-21 almost completely abrogated early virus-specific antibody production, indicating that both cytokines cooperate to drive plasma cell responses (Figure 4). IL-6 is a potent growth and maturation factor for developing human plasma cells, both in vitro [54], [55] and in vivo (including pathogenic conditions [56], [57], [58], [59]), but has minimal capacity to directly induce plasma cell differentiation [54], [60]. IL-6 enhances the proliferation and survival of plasmablasts [54], B cells that have already initiated the process of differentiating to plasma cells. IL-6 also enhances the antibody secretion of those plasmablasts [61]. Given that IL-6 signals through STAT3 in B cells and can upregulate Blimp-1 expression [47], [61], it is likely that IL-6 enhances immunoglobulin production of developing plasma cells by maximizing Blimp-1 expression [61], which consequently upregulates the massive secretory apparatus program of plasma cells [47], [62]. Interestingly, this role of IL-6 appears to be primarily important early during a T-dependent immune response, as antibody levels in the absence of IL-6 are normal by day 15 post-infection [27]. This indicates that IL-6 is predominantly involved in enhancing the maturation of T-dependent extrafollicular plasma cells, prior to germinal center formation. Germinal center formation is detectable at day 6 after acute LCMV infection, with a large germinal center response present by day 8 post-infection. The reduction in plasma cell numbers at day 8 in the absence of IL-21 is most likely a reflection of the critical role of IL-21 in driving plasma cell differentiation from germinal center B cells (Figure 4B–C) [25], [26], [46]. IL-21 is a potent STAT3-dependent inducer of Blimp-1 [40], [47], [48]. Thus the combined absence of IL-6 and IL-21 through 8 days post-infection results in a predominantly IL-6 dependent loss of antibody production by the extrafollicular plasma cells, and the absence of germinal center derived plasma cells (IL-21 dependent), resulting in a near complete abrogation of the serum antibody response to the viral infection.

We observed no early role for IL-6 in germinal center B cell differentiation. The combined loss of both IL-21 and IL-6 had no effect on GC B cell frequency beyond IL-21 deficiency alone (Figure 5). Therefore, enhancement of germinal center B cell proliferation is a unique attribute of IL-21. IL-6 may function solely to enhance plasma cell maturation and antibody production. Stromal cells in the B cell follicle, most likely follicular dendritic cells, are capable of producing IL-6 [3], [63], [64]. However, IL-21 is not the sole factor involved in GC B cell differentiation and proliferation, as the germinal center response in the absence of IL-21 was approximately half of wild type levels. IL-21R^−/−^IL-4^−/−^ mice exhibit a more severe loss of GC B cells than IL-21R^−/−^ mice [44]. In addition to being a class switch factor, IL-4 enhances B cell survival and proliferation [65], [66]. Interestingly, IL-4 is specifically upregulated in GC Tfh cells [17], [67], [68], [69]. IL-4 secretion by GC Tfh is controlled by a different pathway than Th2 produced IL-4 [17]. It is conceivable that numerous soluble factors can contribute significantly to germinal center B cell differentiation and maintenance, given that affinity maturation and the generation of germinal center derived plasma cells and memory B cells is a central process of adaptive immunity. This is an area worthy of extensive further investigation.

The combined absence of IL-6 and IL-21 led to a significant reduction in Tfh frequency, with a corresponding reduction in both CXCR5 and Bcl6 protein expression (Figure 6). In contrast, no effect on Tfh differentiation was observed with the loss of IL-6 alone or IL-21 alone (Figure 6), suggesting that these cytokines share redundant functions in vivo in regulating development of Tfh in response to a viral infection. Exactly how IL-6 and IL-21 regulate Tfh development is still unclear. The reduction in Bcl6 protein levels in Tfh that developed in the combined absence of IL-6 and IL-21 indicates these cytokines have a direct effect on the Bcl6 gene in CD4 T cells in vivo. This is consistent with in vitro data on the effects of IL-6 and IL-21 (Figure 3 and ref. [5]).

Tfh development and function is intimately linked with B cells through their co-localization and selective upregulation of complementary surface molecules that enhance T-B interactions [4], [8], [17], [20], [70], and Tfh are not normally observed in the absence of antigen-specific interactions with B cells [4], [12], [68], [71]. Constitutive expression of Bcl6 in CD4 T cells can overcome the requirement for cognate T-B interactions during Tfh development, indicating that B cells provide a signal inducing or enhancing Bcl6 expression in CD4 T cells [4], [20]. In this context, it is interesting to note that Tfh development was largely unaffected in IL-21^−/−^ mice, which had reduced germinal center B cells compared to wildtype mice (Figure 6). T cell help is commonly the limiting factor in germinal center B cell numbers [72], and IL-21 production is an important part of the T cell help. While T cell help is limiting for germinal center B cell numbers, the converse is not the case here. Tfh depend on the presence of cognate B cells [4], but the IL-21^−/−^ data here leads to the reasonable assumption that cognate B cells are required but not limiting for Tfh numbers at the peak of the antiviral CD4 T cell response. Furthermore, since germinal center development was equivalent in IL-21^−/−^ mice and IL-21^−/−^ mice treated with anti-IL-6 neutralizing mAb, the reduced Tfh numbers in the combined absence of both cytokines is interpreted as a direct effect on the CD4 T cells, not via germinal center B cells.

Curiously, IL-21 [40] and IL-6 (Figure 3) both induce Blimp-1 in purified naive CD4 T cells in vitro. Blimp-1 blocks Tfh differentiation [4]. This indicates that an additional signaling pathway, independent of IL-6 or IL-21, must be activated to prevent Blimp-1 expression and allow for Bcl6 expression to predominate. Furthermore, while IL-6 and IL-21 clearly participate in generation of the Tfh subset, substantial Tfh differentiation was observed in the absence of both cytokines. This indicates Tfh differentiation pathways exist that are IL-6 and IL-21 independent.

Cytokine receptor engagement can induce upregulation of the JAK-STAT pathway. Both IL-6 and IL-21 signal predominantly through STAT3 [28], [31], [40], [43], [73], although STAT1 and STAT5 are also activated in the presence of these cytokines [74]. It has been reported that STAT3^fl/fl^ CD4-Cre^+^ mice exhibit reduced Tfh frequencies, though only a single mouse was shown [7]. In B cells, the role of STAT3 is complex. STAT3 is important in B cells for the induction of Blimp1 [40] and plasma cell differentiation [48], [75]. However, STAT3 can also induce Bcl6 in B cells [46]. Nevertheless, STAT3 is not required for Bcl6 expression in B cells and germinal center formation, as mice or humans with STAT3 deficiency have germinal centers [46], [75]. IL-6 and IL-21 induce both Bcl6 and Blimp-1 in CD4 T cells. STAT3^fl/fl^ CD4-Cre^+^ CD4 T cells fail to upregulate Blimp-1 upon TCR stimulation in the presence of IL-21 [40]. These findings indicate that the roles of STAT3 in both CD4 T cells and B cells may be complex, and may not directly correlate with the functions of IL-6 and IL-21.

In the context of an acute viral infection, we observed complete redundancy in the roles for IL-6 and IL-21 in Tfh development at the peak of the effector CD4 T cell response. Redundancies in signaling pathways and cellular differentiation processes are frequently associated with crucial biological processes. Therefore, it is not surprising that there is a high degree of overlap in signals needed for generation of the CD4 T cells required for B cell help, given that antibody responses are essential for combating a vast array of infectious diseases. In addition, redundancies in pathways also make it substantially more difficult for pathogens to evade the immune system. IL-6 and IL-21 are largely produced by different cell types (DCs and non-hematopoeitic cells vs. CD4 T cells) and are dependent upon the stimuli encountered. Therefore, it may be expected that, of IL-6 or IL-21, one likely has a more prominent role than the other in the context of different antigenic insults at different sites under different conditions, or if a pathogen suppresses induction of one of these cytokines.

While our data support a collaborative role for IL-6 and IL-21 in Tfh differentiation, these cytokines are not sufficient for instruction of Tfh differentiation (Figure 3). All of the other known CD4 T cell subsets can be derived from purified naïve CD4 T cells cultured in the presence of cytokine(s) plus TCR stimulation [76], and these polarized cells express high levels of their signature cytokine as well as upregulation of their associated master regulator transcription factor. However, here we have demonstrated that induction of high IL-21 from polarized naive purified CD4 T cell cultures (Figure 2) does not equal Tfh differentiation, as characterized by increased protein expression of both Bcl6 and CXCR5 (Figure 3). IL-6 or IL-21 are therefore insufficient to drive Tfh differentiation in vitro. Alternatively, Tfh may require an additional cytokine for their induction, in keeping with the mechanism of other CD4 T cell differentiation pathways. This observation does not contradict earlier studies in which in vitro Tfh or pre-Tfh were reported [5], [77]. Those studies utilized naïve CD4 T cells cultured with irradiated splenocytes supplemented with either IL-6 or IL-21. We interpret all of these results as supporting the conclusion that IL-6 and IL-21 are insufficient, but they can interact with additional factors present in vivo, or in more complex cell mixtures in vitro, to drive Tfh differentiation.

Controversy has surrounded the role of IL-21 and IL-6 in Tfh differentiation, as the absence of either cytokine alone has often led to conflicting reports of Tfh development in vivo [7], [22], [24], [25], [26], [27]. This study is the first to characterize the effects on Tfh differentiation and B cell development in the combined absence of both IL-6 and IL-21. We demonstrate that these cytokines share cooperative functions in the generation of humoral immunity in vivo. Tfh differentiation is a complex process with multiple overlapping and redundant signals promoting the development of this subset. These results help clarify the path to understanding the signals necessary and sufficient for Tfh differentiation.

## References

[1] Crotty S, Ahmed R (2004). Immunological memory in humans.. Semin Immunol.

[2] MacLennan IC (1994). Germinal centers.. Annu Rev Immunol.

[3] Allen CDC, Okada T, Cyster JG (2007). Germinal-center organization and cellular dynamics.. Immunity.

[4] Johnston RJ, Poholek AC, DiToro D, Yusuf I, Eto D (2009). Bcl6 and Blimp-1 are reciprocal and antagonistic regulators of T follicular helper cell differentiation.. Science.

[5] Nurieva RI, Chung Y, Martinez GJ, Yang XO, Tanaka S (2009). Bcl6 mediates the development of T follicular helper cells.. Science.

[6] Yu D, Rao S, Tsai LM, Lee SK, He Y (2009). The transcriptional repressor Bcl-6 directs T follicular helper cell lineage commitment.. Immunity.

[7] Nurieva RI, Chung Y, Hwang D, Yang XO, Kang HS (2008). Generation of T follicular helper cells is mediated by interleukin-21 but independent of T helper 1, 2, or 17 cell lineages.. Immunity.

[8] Crotty S (2011). Follicular Helper CD4 T Cells (T(FH))..

[9] Schaerli P, Willimann K, Lang AB, Lipp M, Loetscher P (2000). CXC chemokine receptor 5 expression defines follicular homing T cells with B cell helper function.. J Exp Med.

[10] Breitfeld D, Ohl L, Kremmer E, Ellwart J, Sallusto F (2000). Follicular B helper T cells express CXC chemokine receptor 5, localize to B cell follicles, and support immunoglobulin production.. J Exp Med.

[11] Kim CH, Rott LS, Clark-Lewis I, Campbell DJ, Wu L (2001). Subspecialization of CXCR5+ T cells: B helper activity is focused in a germinal center-localized subset of CXCR5+ T cells.. J Exp Med.

[12] Haynes NM, Allen CDC, Lesley R, Ansel KM, Killeen N (2007). Role of CXCR5 and CCR7 in follicular Th cell positioning and appearance of a programmed cell death gene-1high germinal center-associated subpopulation.. J Immunol.

[13] Hardtke S, Ohl L, Förster R (2005). Balanced expression of CXCR5 and CCR7 on follicular T helper cells determines their transient positioning to lymph node follicles and is essential for efficient B-cell help.. Blood.

[14] Förster R, Mattis AE, Kremmer E, Wolf E, Brem G (1996). A putative chemokine receptor, BLR1, directs B cell migration to defined lymphoid organs and specific anatomic compartments of the spleen.. Cell.

[15] Akiba H, Takeda K, Kojima Y, Usui Y, Harada N (2005). The role of ICOS in the CXCR5+ follicular B helper T cell maintenance in vivo.. J Immunol.

[16] Rasheed A-U, Rahn H-P, Sallusto F, Lipp M, Müller G (2006). Follicular B helper T cell activity is confined to CXCR5hiICOShi CD4 T cells and is independent of CD57 expression.. Eur J Immunol.

[17] Yusuf I, Kageyama R, Monticelli L, Johnston RJ, Ditoro D (2010). Germinal Center T Follicular Helper Cell IL-4 Production Is Dependent on Signaling Lymphocytic Activation Molecule Receptor (CD150).. The Journal of Immunology.

[18] Vinuesa CG, Tangye SG, Moser B, Mackay CR (2005). Follicular B helper T cells in antibody responses and autoimmunity.. Nat Rev Immunol.

[19] King C, Tangye SG, Mackay CR (2008). T follicular helper (TFH) cells in normal and dysregulated immune responses.. Annu Rev Immunol.

[20] Crotty S, Johnston RJ, Schoenberger SP (2010). Effectors and memories: Bcl-6 and Blimp-1 in T and B lymphocyte differentiation.. Nat Immunol.

[21] Suto A, Kashiwakuma D, Kagami S-i, Hirose K, Watanabe N (2008). Development and characterization of IL-21-producing CD4+ T cells.. J Exp Med.

[22] Vogelzang A, McGuire HM, Yu D, Sprent J, Mackay CR (2008). A fundamental role for interleukin-21 in the generation of T follicular helper cells.. Immunity.

[23] Chtanova T, Tangye SG, Newton R, Frank N, Hodge MR (2004). T follicular helper cells express a distinctive transcriptional profile, reflecting their role as non-Th1/Th2 effector cells that provide help for B cells.. J Immunol.

[24] Yi JS, Du M, Zajac AJ (2009). A vital role for interleukin-21 in the control of a chronic viral infection.. Science.

[25] Linterman MA, Beaton L, Yu D, Ramiscal RR, Srivastava M (2010). IL-21 acts directly on B cells to regulate Bcl-6 expression and germinal center responses.. J Exp Med.

[26] Zotos D, Coquet JM, Zhang Y, Light A, D'Costa K (2010). IL-21 regulates germinal center B cell differentiation and proliferation through a B cell-intrinsic mechanism.. J Exp Med.

[27] Poholek AC, Hansen K, Hernandez SG, Eto D, Chandele A (2010). In vivo regulation of bcl6 and T follicular helper cell development.. J Immunol.

[28] Eddahri F, Denanglaire S, Bureau F, Spolski R, Leonard WJ (2009). Interleukin-6/STAT3 signaling regulates the ability of naive T cells to acquire B-cell help capacities.. Blood.

[29] Nurieva R, Yang XO, Martinez G, Zhang Y, Panopoulos AD (2007). Essential autocrine regulation by IL-21 in the generation of inflammatory T cells.. Nature.

[30] Korn T, Bettelli E, Gao W, Awasthi A, Jäger A (2007). IL-21 initiates an alternative pathway to induce proinflammatory TH17 cells.. Nature.

[31] Wei L, Laurence A, Elias KM, O'Shea JJ (2007). IL-21 is produced by Th17 cells and drives IL-17 production in a STAT3-dependent manner.. J Biol Chem.

[32] Spolski R, Leonard WJ (2008). Interleukin-21: basic biology and implications for cancer and autoimmunity.. Annu Rev Immunol.

[33] Dienz O, Eaton S, Bond J, Neveu W, Moquin D (2009). The induction of antibody production by IL-6 is indirectly mediated by IL-21 produced by CD4+ T cells.. J Exp Med.

[34] Dienz O, Rincon M (2009). The effects of IL-6 on CD4 T cell responses.. Clinical Immunology.

[35] Kopf M, Baumann H, Freer G, Freudenberg M, Lamers M (1994). Impaired immune and acute-phase responses in interleukin-6-deficient mice.. Nature.

[36] McCausland MM, Yusuf I, Tran H, Ono N, Yanagi Y (2007). SAP regulation of follicular helper CD4 T cell development and humoral immunity is independent of SLAM and Fyn kinase.. J Immunol.

[37] Ma CS, Suryani S, Avery DT, Chan A, Nanan R (2009). Early commitment of naïve human CD4(+) T cells to the T follicular helper (T(FH)) cell lineage is induced by IL-12.. Immunol Cell Biol.

[38] Martins GA, Cimmino L, Shapiro-Shelef M, Szabolcs M, Herron A (2006). Transcriptional repressor Blimp-1 regulates T cell homeostasis and function.. Nat Immunol.

[39] Ozaki K, Spolski R, Ettinger R, Kim H-P, Wang G (2004). Regulation of B cell differentiation and plasma cell generation by IL-21, a novel inducer of Blimp-1 and Bcl-6.. J Immunol.

[40] Kwon H, Thierry-Mieg D, Thierry-Mieg J, Kim H-P, Oh J (2009). Analysis of interleukin-21-induced Prdm1 gene regulation reveals functional cooperation of STAT3 and IRF4 transcription factors.. Immunity.

[41] Allman D, Jain A, Dent A, Maile RR, Selvaggi T (1996). BCL-6 expression during B-cell activation.. Blood.

[42] Saito Y, Liang G, Egger G, Friedman JM, Chuang JC (2006). Specific activation of microRNA-127 with downregulation of the proto-oncogene BCL6 by chromatin-modifying drugs in human cancer cells.. Cancer Cell.

[43] Heinrich PC, Behrmann I, Haan S, Hermanns HM, Müller-Newen G (2003). Principles of interleukin (IL)-6-type cytokine signalling and its regulation.. Biochem J.

[44] Ozaki K, Spolski R, Feng CG, Qi C-F, Cheng J (2002). A critical role for IL-21 in regulating immunoglobulin production.. Science.

[45] Crotty S, Kersh EN, Cannons J, Schwartzberg PL, Ahmed R (2003). SAP is required for generating long-term humoral immunity.. Nature.

[46] Avery DT, Deenick EK, Ma CS, Suryani S, Simpson N (2010). B cell-intrinsic signaling through IL-21 receptor and STAT3 is required for establishing long-lived antibody responses in humans.. J Exp Med.

[47] Martins G, Calame K (2008). Regulation and functions of Blimp-1 in T and B lymphocytes.. Annu Rev Immunol.

[48] Diehl SA, Schmidlin H, Nagasawa M, van Haren SD, Kwakkenbos MJ (2008). STAT3-mediated up-regulation of BLIMP1 Is coordinated with BCL6 down-regulation to control human plasma cell differentiation.. J Immunol.

[49] Elsaesser H, Sauer K, Brooks DG (2009). IL-21 is required to control chronic viral infection.. Science.

[50] Diehl S, Anguita J, Hoffmeyer A, Zapton T, Ihle JN (2000). Inhibition of Th1 differentiation by IL-6 is mediated by SOCS1.. Immunity.

[51] Suto A, Wurster AL, Reiner SL, Grusby MJ (2006). IL-21 inhibits IFN-gamma production in developing Th1 cells through the repression of Eomesodermin expression.. J Immunol.

[52] Ettinger R, Sims GP, Fairhurst A-M, Robbins R, da Silva YS (2005). IL-21 induces differentiation of human naive and memory B cells into antibody-secreting plasma cells.. J Immunol.

[53] Bryant VL, Ma CS, Avery DT, Li Y, Good KL (2007). Cytokine-mediated regulation of human B cell differentiation into Ig-secreting cells: predominant role of IL-21 produced by CXCR5+ T follicular helper cells.. J Immunol.

[54] Jego G, Bataille R, Pellat-Deceunynck C (2001). Interleukin-6 is a growth factor for nonmalignant human plasmablasts.. Blood.

[55] Rousset F, Garcia E, Banchereau J (1991). Cytokine-induced proliferation and immunoglobulin production of human B lymphocytes triggered through their CD40 antigen.. J Exp Med.

[56] Jego G, Robillard N, Puthier D, Amiot M, Accard F (1999). Reactive plasmacytoses are expansions of plasmablasts retaining the capacity to differentiate into plasma cells.. Blood.

[57] Bataille R, Barlogie B, Lu ZY, Rossi JF, Lavabre-Bertrand T (1995). Biologic effects of anti-interleukin-6 murine monoclonal antibody in advanced multiple myeloma.. Blood.

[58] Klein B, Zhang XG, Lu ZY, Bataille R (1995). Interleukin-6 in human multiple myeloma.. Blood.

[59] van Zaanen HC, Koopmans RP, Aarden LA, Rensink HJ, Stouthard JM (1996). Endogenous interleukin 6 production in multiple myeloma patients treated with chimeric monoclonal anti-IL6 antibodies indicates the existence of a positive feed-back loop.. J Clin Invest.

[60] Jego G, Palucka AK, Blanck JP, Chalouni C, Pascual V (2003). Plasmacytoid dendritic cells induce plasma cell differentiation through type I interferon and interleukin 6.. Immunity.

[61] González-García I, Ocaña E, Jiménez-Gómez G, Campos-Caro A, Brieva JA (2006). Immunization-induced perturbation of human blood plasma cell pool: progressive maturation, IL-6 responsiveness, and high PRDI-BF1/BLIMP1 expression are critical distinctions between antigen-specific and nonspecific plasma cells.. J Immunol.

[62] Shapiro-Shelef M, Calame K (2005). Regulation of plasma-cell development.. Nat Rev Immunol.

[63] Kopf M, Herren S, Wiles MV, Pepys MB, Kosco-Vilbois MH (1998). Interleukin 6 influences germinal center development and antibody production via a contribution of C3 complement component.. J Exp Med.

[64] Wu Y, El Shikh MEM, El Sayed RM, Best AM, Szakal AK (2009). IL-6 produced by immune complex-activated follicular dendritic cells promotes germinal center reactions, IgG responses and somatic hypermutation.. Int Immunol.

[65] Vitetta ES, Ohara J, Myers CD, Layton JE, Krammer PH (1985). Serological, biochemical, and functional identity of B cell-stimulatory factor 1 and B cell differentiation factor for IgG1.. J Exp Med.

[66] Howard M, Farrar J, Hilfiker M, Johnson B, Takatsu K (1982). Identification of a T cell-derived b cell growth factor distinct from interleukin 2.. J Exp Med.

[67] Reinhardt RL, Liang H-E, Locksley RM (2009). Cytokine-secreting follicular T cells shape the antibody repertoire.. Nat Immunol.

[68] Zaretsky AG, Taylor JJ, King IL, Marshall FA, Mohrs M (2009). T follicular helper cells differentiate from Th2 cells in response to helminth antigens.. J Exp Med.

[69] King IL, Mohrs M (2009). IL-4-producing CD4+ T cells in reactive lymph nodes during helminth infection are T follicular helper cells.. J Exp Med.

[70] Qi H, Cannons JL, Klauschen F, Schwartzberg PL, Germain RN (2008). SAP-controlled T-B cell interactions underlie germinal centre formation.. Nature.

[71] Deenick EK, Chan A, Ma CS, Gatto D, Schwartzberg PL (2010). Follicular helper T cell differentiation requires continuous antigen presentation that is independent of unique B cell signaling.. Immunity.

[72] Rolf J, Bell SE, Kovesdi D, Janas ML, Soond DR (2010). Phosphoinositide 3-kinase activity in T cells regulates the magnitude of the germinal center reaction.. J Immunol.

[73] Zhou L, Ivanov II, Spolski R, Min R, Shenderov K (2007). IL-6 programs T(H)-17 cell differentiation by promoting sequential engagement of the IL-21 and IL-23 pathways.. Nat Immunol.

[74] Hotson AN, Hardy JW, Hale MB, Contag CH, Nolan GP (2009). The T cell STAT signaling network is reprogrammed within hours of bacteremia via secondary signals.. J Immunol.

[75] Fornek JL, Tygrett LT, Waldschmidt TJ, Poli V, Rickert RC (2006). Critical role for Stat3 in T-dependent terminal differentiation of IgG B cells.. Blood.

[76] Zhu J, Yamane H, Paul WE (2010). Differentiation of effector CD4 T cell populations (*).. Annu Rev Immunol.

[77] Kashiwakuma D, Suto A, Hiramatsu Y, Ikeda K, Takatori H (2010). B and T lymphocyte attenuator suppresses IL-21 production from follicular Th cells and subsequent humoral immune responses.. J Immunol.

